# Paired Inter-Lesion Invasiveness and Recurrence Risk in Synchronous Multiple Primary Lung Adenocarcinoma: Focus on the IAC-Dominant Subgroup

**DOI:** 10.3390/jcm15187317

**Published:** 2026-09-20

**Authors:** Xueyu Chen, Tong Lu, Dong Dong, Yajie Zhang, Hecheng Li

**Affiliations:** Department of Thoracic Surgery, Ruijin Hospital, Shanghai Jiao Tong University School of Medicine, Shanghai 200025, China

**Keywords:** multiple primary lung cancer, lung adenocarcinoma, invasiveness concordance, synchronous lesions, PD-L1, spread through air spaces, disease-free survival, nomogram, Firth regression

## Abstract

**Background:** Whether paired invasiveness patterns, particularly when both lesions are invasive adenocarcinoma (IAC + IAC), carry prognostic value beyond single-lesion staging in synchronous multiple primary lung adenocarcinoma (SMPLA) remains unclear. We assessed preoperative predictors of concordance, its underlying clinicopathological aggressiveness gradient, and its impact on disease-free survival (DFS). **Methods:** We retrospectively analyzed 198 patients with two SMPLA lesions, classified as concordant (match, *n* = 104) or discordant (mismatch, *n* = 94) based on identical versus differing AIS/MIA/IAC invasiveness grade. Preoperative predictors of mismatch were assessed by multivariable logistic regression. A pre-specified WHO-based paired invasiveness score was used to test trends in six aggressiveness markers via Cochran–Armitage tests. Within an IAC-dominant subset (Lesion 1 = IAC, *n* = 91), Firth-penalized Cox regression assessed the effect of Lesion 2 subtype (IAC vs. AIS/MIA) on DFS. **Results:** Same-lobe location was more frequent in the mismatch group (75.5% vs. 58.7%, *p* = 0.016), and Lesion 1 PD-L1 TPS was lower (0.8% vs. 1.0%, *p* = 0.005). On multivariable analysis, same-lobe location (adjusted OR = 2.26, 95% CI 1.16–4.41, *p* = 0.017) and Lesion 2 size (adjusted OR = 0.79/mm, *p* < 0.001) independently predicted mismatch (AUC = 0.671); adding PD-L1 status gave no incremental discrimination (*p* = 0.119). Across the paired invasiveness score, five of six markers—PD-L1 TPS ≥ 1%, Ki-67 ≥ 10%, Ki-67 ≥ 20%, STAS, and pleural invasion—showed significant increasing trends (all *p* ≤ 0.020), while vascular invasion did not (*p* = 0.113). Within the IAC-dominant subset, the adverse recurrence signal was observed specifically in the IAC + IAC group (5/34 [14.7%] vs. 1/57 [1.8%] events; HR = 5.28, 95% CI 1.04–51.84, *p* = 0.044). This finding should be interpreted as hypothesis-generating rather than confirmatory, given only six total events and a wide confidence interval. **Conclusions:** In high-risk SMPLA, paired-lesion risk assessment—particularly the distinction between IAC + IAC and IAC + AIS/MIA pairs—may complement single-lesion staging, pending prospective validation.

## 1. Introduction

Multiple primary lung cancer (MPLC) is being diagnosed with increasing frequency, driven by the widespread adoption of low-dose computed tomography screening, with recent series reporting synchronous MPLC (sMPLC) in up to 20% of lung cancer patients, though estimates vary widely depending on diagnostic criteria and screening intensity [1,2,3]. Among sMPLC presentations, synchronous double primary lung cancer (sDPLC) is the predominant clinical subtype, accounting for 75.35% (269/357) of sMPLC cases in a large Chinese cohort [4]. Given its predominance, findings from sDPLC therefore bear directly on sMPLC as a whole; the present study focuses on synchronous multiple primary lung adenocarcinoma (SMPLA), the most common histological configuration within sDPLC.

Among patients with SMPLA, recurrence risk and prognosis are inherently complex and not adequately captured by single-lesion staging alone, as reflected in the substantial heterogeneity of outcomes reported across MPLC cohorts and the absence of standardized, biologically grounded risk-stratification frameworks [5]. The presence of a second lesion—even when indolent—introduces additional dimensions of heterogeneity: the two lesions may differ in histological subtype, molecular profile, and biological aggressiveness, yet current staging algorithms assign the overall stage based solely on the most advanced lesion, without formal accounting for the pathological relationship between paired tumors [6]. Studies have shown that molecular driver mutations can be highly discordant between synchronous lesions within the same patient, and that this inter-lesion heterogeneity may itself carry prognostic information beyond either lesion analyzed in isolation [7,8,9].

Despite this recognition, comparatively little is known about how histological invasiveness—the internationally adopted adenocarcinoma in situ (AIS), minimally invasive adenocarcinoma (MIA), and invasive adenocarcinoma (IAC) classification [10]—varies between paired lesions, and whether concordance or discordance in invasiveness grade carries independent prognostic significance. More recently, Qiu et al. demonstrated that concurrent spread through air spaces (STAS) affecting both dominant tumors independently predicted worse survival in SMPLA, illustrating that pathological features assessed jointly across paired lesions—rather than in either lesion alone—can reveal prognostic information not captured by single-lesion analysis [11]. However, whether the paired histological invasiveness relationship itself constitutes an informative prognostic axis has not been systematically examined.

In this study, we retrospectively analyzed 198 patients with SMPLA to characterize the relationship between paired lesion invasiveness—classified as histologically matched (concordant AIS/MIA/IAC) versus mismatched (discordant)—and clinicopathological, immunohistochemical, and survival outcomes. Specifically, we (i) identified preoperative correlates of pathological mismatch using logistic regression, (ii) examined whether tumor aggressiveness biomarkers exhibited a monotonic gradient across a pre-specified paired invasiveness score, and (iii) evaluated whether the secondary lesion’s histological identity carried prognostic value for disease-free survival within the IAC-dominant subgroup. These analyses aim to clarify whether within-patient histological concordance provides prognostic information beyond conventional single-lesion staging.

## 2. Methods

### 2.1. Study Design and Population

This retrospective cohort study was conducted at the Department of Thoracic Surgery, Ruijin Hospital, Shanghai Jiao Tong University School of Medicine. The protocol was approved by the Ethics Committee of Ruijin Hospital (No. Ruijin-2021[219]). Informed consent was taken from all the participants. Consecutive patients who underwent surgical resection of pulmonary lesions between January 2019 and December 2023 were screened.

Inclusion criteria were: (1) surgical resection at our department; (2) postoperative pathological diagnosis of AIS, MIA, or IAC; (3) synchronous MPLC confirmed by histopathological, immunohistochemical, and clinical criteria; (4) unambiguous determination of lesion number, location, and histological subtype from pathology reports; and (5) complete clinicopathological data for the planned analyses. Exclusion criteria were: prior malignancy, evidence of intrapulmonary metastasis rather than MPLC, non-adenocarcinoma histology, missing key data, or absence of surgical resection. After applying these criteria, 198 patients were included.

### 2.2. Diagnostic Criteria for SMPLA

SMPLA was diagnosed according to the 2013 ACCP evidence-based clinical practice guidelines on the diagnosis and management of SMPLA [6], confirmed by a multidisciplinary team integrating histopathology, immunohistochemistry, and radiology. The distinction between SMPLA and intrapulmonary metastasis is relevant only for IAC–IAC pairs, as lesion pairs with lower-grade components cannot represent metastases. For same-lobe IAC–IAC pairs (*n* = 3), clonal independence was confirmed by next-generation sequencing. Different-lobe IAC–IAC pairs (*n* = 31) were classified as independent primaries according to ACCP criteria, which consider same-lobe location a major risk factor for metastasis; this approach is consistent with standard clinical practice, though NGS-based confirmation was not performed in these cases due to cost considerations.

### 2.3. Definitions of Lesions and Match/Mismatch Groups

For paired-lesion analysis, a consistent hierarchical reference was established by defining Lesion 1 as the more invasive lesion (or the larger if invasiveness was equal), with Lesion 2 as the secondary lesion. This definition enabled systematic characterization of all 198 patient pairs across diverse histological combinations (e.g., IAC + MIA, AIS + MIA, MIA + MIA).

This definition necessarily creates a structural association between Lesion 1 characteristics and the match/mismatch classification in full-cohort analyses—for example, all mismatch cases have an IAC-dominant Lesion 1 by construction. Full-cohort comparisons involving Lesion 1 features should therefore be interpreted as descriptive characterizations of concordance patterns rather than independent biological discoveries. In contrast, the IAC-dominant subset analysis (Lesion 1 uniformly IAC) eliminates this dependency and is appropriate for prognostic inference.

Patients were classified as **match** (both lesions sharing identical invasiveness category, e.g., IAC + IAC, MIA + MIA, AIS + AIS) or **mismatch** (discordant categories, e.g., IAC + MIA). This yielded 104 match and 94 mismatch patients.

### 2.4. Data Collection

Demographic, smoking history, radiological (location, lobe), and pathological (size, histological subtype, T category, nodal status) variables were abstracted from medical records. Surgical resection type (lobectomy, segmentectomy, or wedge resection) was determined intraoperatively based on frozen-section pathology and lesion characteristics. All resections achieved microscopically negative margins (R0).

### 2.5. Immunohistochemistry

Ki-67 was stained with the MIB-1 antibody and scored as the percentage of positive tumor cells in the hot spot; cutoffs of ≥10% and ≥20% were applied for stratification, consistent with cutoff ranges reported in a systematic review of Ki-67’s clinical significance in NSCLC [12]. PD-L1 expression was assessed with the 22C3 pharmDx assay, and Tumor Proportion Score (TPS) was categorized as <1% (negative) or ≥1% (positive), consistent with standard companion diagnostic scoring criteria [13]. Ki-67 and PD-L1 testing were performed at the discretion of the treating pathology team based on clinical indication and tissue adequacy, rather than as a uniform protocol for all patients; consequently, data were available for 97/198 (49.0%) and 92/198 (46.5%) patients for Ki-67 and PD-L1, respectively. Analyses involving these biomarkers were restricted to patients with available data (complete-case analysis).

### 2.6. STAS and Pleural Invasion

STAS was defined per the 2015 World Health Organization (WHO) classification as micropapillary clusters, solid nests, or single tumor cells present within air spaces beyond the edge of the main tumor, and was assessed on formalin-fixed paraffin-embedded sections by a single thoracic pathologist blinded to clinical outcomes [14]. Visceral pleural invasion was defined as tumor cells breaching the elastic layer of the visceral pleura, identified with elastic staining when required [15]. Both features were recorded as binary variables (present vs. absent) by the same pathologist.

### 2.7. Follow-Up and Outcome Definitions

Postoperative follow-up included chest CT, lymph node ultrasonography, and brain MRI every 4–6 months for the first two years. DFS was defined as the time from surgery to the first recurrence (locoregional or distant) or death from any cause, whichever occurred first; overall survival (OS) was defined as the time from surgery to death from any cause [16]. Patients without an event were censored at the date of last follow-up.

### 2.8. Statistical Analysis

Continuous variables are presented as the median [IQR] and compared by the Mann–Whitney U test (normality assessed by the Shapiro–Wilk test). Categorical variables are compared by Fisher’s exact test or the χ^2^ test, as appropriate. All tests were two-sided; *p* < 0.05 was considered significant. Analyses used Python version 3.12.4 (statsmodels, scipy, lifelines, scikit-learn) and R version 4.3 (coxphf package version 1.13.4).

**Preoperative correlates of mismatch.** A preoperative logistic regression model was built. Approximately 34 candidate variables (Table 1) were screened univariably; those with *p* < 0.10 were advanced to multivariable modeling. Lesion 1 T stage was excluded a priori because its categories overlap definitionally with the histological classification used to define mismatch (incorporation bias, per PROBAST guidance) [17], and Lesion 1 lobe was excluded owing to category multiplicity. Age and sex were removed after showing no discriminative contribution individually or jointly (likelihood-ratio test), yielding a final three-variable model: same-lobe location, Lesion 1 size, and Lesion 2 size. Model performance was assessed by the area under the receiver operating characteristic curve (AUC); overall model significance was tested against an intercept-only null model. Discrimination was further characterized by sensitivity and specificity, calibration was assessed by the Hosmer–Lemeshow test, and internal validation was performed by bootstrap resampling (1000 iterations) to estimate the optimism-corrected AUC. Unadjusted odds ratios from univariable analysis used one-vs-rest coding.

**PD-L1 sensitivity analysis.** In the subset with available PD-L1 data (*n* = 92), the three-variable preoperative model was compared with a nested model additionally containing Lesion 1 PD-L1 TPS ≥ 1%, using the likelihood-ratio test and DeLong’s test for correlated ROC curves [18].

**Clinicopathological aggressiveness gradient across paired invasiveness scores.** Lesion pairs were ordered using a pre-specified summed invasiveness score derived from the WHO/International Association for the Study of Lung Cancer (IASLC) histological grading of pulmonary adenocarcinoma (AIS = 0, MIA = 1, IAC = 2; total range 0–4) [10,19]. The Cochran–Armitage trend test evaluated linear trends across this ordinal score for six aggressiveness markers: PD-L1 TPS ≥ 1%, Ki-67 ≥ 10%, Ki-67 ≥ 20%, STAS, pleural invasion, and vascular invasion. Tied scores (MIA-MIA and AIS-IAC, both score = 2) received equal rank.

**Survival analysis in the IAC-dominant subset.** In the 91 patients with Lesion 1 = IAC, DFS was compared between the IAC + IAC and IAC + AIS/MIA groups using the Kaplan–Meier method and log-rank test. Because only 6 DFS events were observed with a highly unbalanced distribution (5 vs. 1), conventional Cox regression was considered unreliable; Firth’s penalized-likelihood Cox regression was applied instead, which provides finite, less-biased estimates in small-sample, rare-event settings [20]. Hazard ratios (HR) and profile-likelihood 95% confidence intervals were estimated from a univariable model containing only the group indicator. Two pre-specified sensitivity analyses each added a single covariate (Lesion 1 T stage, ordinal; or Lesion 1 size, continuous) to assess potential confounding by dominant-lesion tumor burden. OS was not modeled owing to a single observed event and is reported descriptively.

## 3. Results

### 3.1. Baseline Characteristics

Among 198 patients with SMPLA, 104 (52.5%) had concordant pathological invasiveness between Lesion 1 and Lesion 2 (match group), while 94 (47.5%) had discordant invasiveness (mismatch group).

Age, sex, and smoking history were well balanced between groups (all *p* > 0.05, Table 2). Same-lobe distribution was markedly more frequent in the mismatch group (75.5% vs. 58.7%, *p* = 0.016), and Lesion 1 size showed a nonsignificant trend toward being larger (10 mm [IQR 7–14] vs. 8 mm [IQR 6–11], *p* = 0.059); the lesion count, nodal status, and lesion location did not differ. The surgical procedure for the dominant lesion was comparable between groups (*p* = 0.658), although the detailed four-category distribution differed significantly (*p* = 0.002), with lobectomy alone being more frequent in the mismatch group (28.7% vs. 10.6%) and lobectomy plus sublobar resection being more frequent in the match group (24.0% vs. 9.6%).

The overall clinical stage and the T-stage/pathological subtype distributions of both lesions differed significantly between groups (all *p* < 0.001). As noted in the Methods Section, these differences reflect the Lesion 1 definition rather than independent biological discoveries and should be interpreted as descriptive characterizations of concordance patterns. Notably, Lesion 1 PD-L1 TPS was higher in the match group (median 1.0% [IQR 0.8–3.5] vs. 0.8% [IQR 0.0–1.0]; *n* = 92; *p* = 0.005), although the absolute difference was small. Ki-67 expression did not differ significantly between groups (*n* = 97; *p* = 0.901), and the dominant histological subtype (predominantly acinar) was similarly distributed (*p* = 0.645).

These systematic differences—particularly the more frequent same-lobe presentation and lower PD-L1 expression in the mismatch group—motivated a formal investigation into the preoperative predictors of pathological mismatch and the potential clinicopathological aggressiveness gradient underlying this dichotomy (Section 3.2 and Section 3.3).

### 3.2. Preoperative Correlates of Pathological Mismatch

Following the variable selection procedure described in the Section 2, the final preoperative model (same-lobe location, Lesion 1 size, Lesion 2 size) significantly outperformed an intercept-only null model (likelihood-ratio test, χ^2^ = 24.06, df = 3, *p* < 0.001) and showed discrimination comparable to the extended five-variable model (AUC = 0.671 vs. 0.673), confirming that dropping age and sex did not meaningfully sacrifice performance.

**Figure 1:**
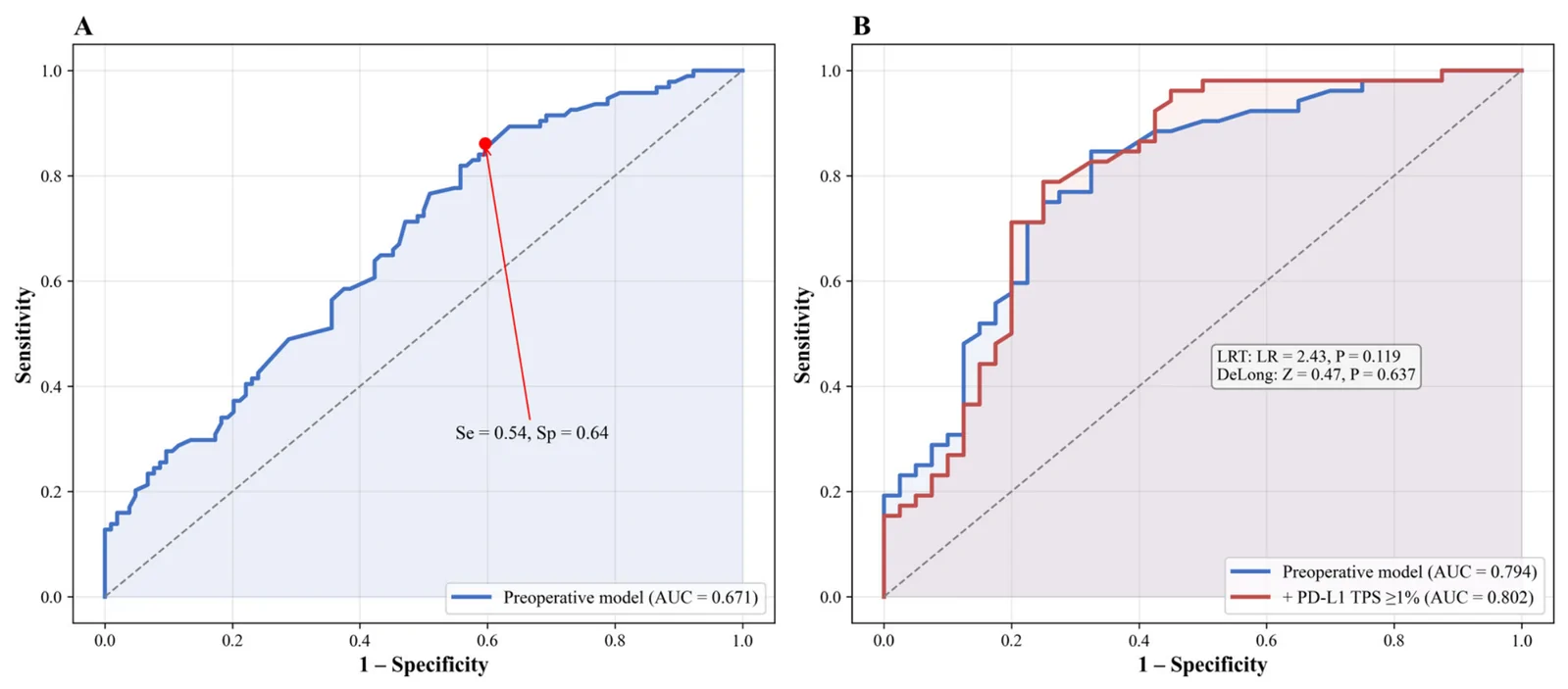
Discriminative performance of the preoperative model for pathological mismatch. (**A**) ROC curve of the primary preoperative model (same-lobe location, Lesion 1 size, Lesion 2 size) fitted on the full cohort (*N* = 198, 94 mismatch events); AUC = 0.671. (**B**) ROC curves comparing the PD-L1-inclusive sensitivity model (same-lobe location, Lesion 1 size, Lesion 2 size, Lesion 1 PD-L1 TPS ≥1%) and the preoperative model (same-lobe location, Lesion 1 size, Lesion 2 size) in the 92-patient subset with available PD-L1 data (52 mismatch events); AUC = 0.802 vs. 0.794, respectively (likelihood-ratio test, LR = 2.43, *p* = 0.119; DeLong test, Z = 0.47, *p* = 0.637).

The model showed an apparent AUC of 0.671, with sensitivity and specificity of 0.543 and 0.644, respectively (Figure 1A). Calibration was adequate by the Hosmer–Lemeshow test (χ^2^ = 4.223, *p* = 0.837). Bootstrap internal validation with 1000 resamples yielded an optimism-corrected AUC of 0.656 (95% CI 0.600–0.748), indicating negligible optimism. These metrics should nonetheless be interpreted as exploratory, as the model has not undergone external validation.

Same-lobe location was independently associated with higher odds of mismatch (adjusted OR = 2.26, 95% CI 1.16–4.41, *p* = 0.017), and larger Lesion 2 size with lower odds (adjusted OR = 0.79 per mm, 95% CI 0.68–0.90, *p* < 0.001; Table 1). Lesion 1 size, which was not univariably significant (*p* = 0.221), became independently associated with mismatch after adjustment for same-lobe status and Lesion 2 size (adjusted OR = 1.14 per mm, 95% CI 1.06–1.22, *p* < 0.001), consistent with a suppression effect whereby the two lesion-size variables jointly captured information that was not evident from either alone.

To determine whether PD-L1 status adds discriminative information beyond these three preoperative variables, a sensitivity analysis was performed in the 92-patient subset with available Lesion 1 PD-L1 data (52 mismatch events), adding PD-L1 TPS ≥ 1% to the three-variable model refit on this subset. This addition did not significantly improve model fit (likelihood-ratio test, LR = 2.43, df = 1, *p* = 0.119), and discrimination was not significantly different from the preoperative model alone (AUC = 0.802 vs. 0.794; DeLong test, Z = 0.47, *p* = 0.637; Figure 1B). PD-L1 status therefore does not provide meaningful incremental value once same-lobe location and lesion size are known, and the complete-cohort preoperative model was retained as primary.

### 3.3. Clinicopathological Aggressiveness Gradient Across WHO-Defined Paired Invasiveness Scores

**Figure 2:**
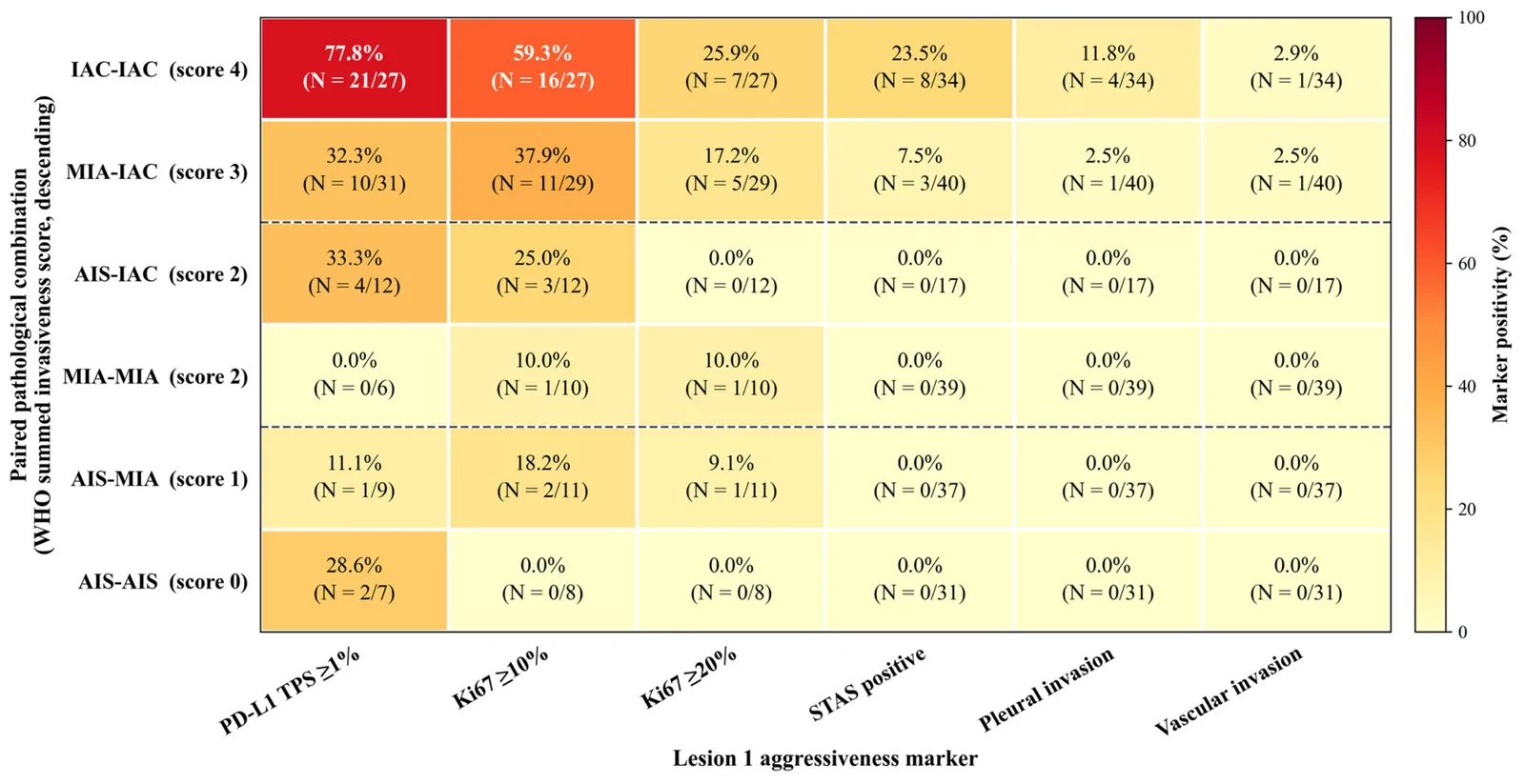
Heatmap of Lesion 1 aggressiveness-marker positivity across six unordered paired pathological combinations, arranged from highest to lowest paired invasiveness score (top to bottom). Combinations are ordered by a pre-specified WHO-based summed invasiveness score, defined as the sum of the single-lesion invasiveness grades (AIS = 0, MIA = 1, IAC = 2; possible total score, 0–4), independent of the marker values displayed in the heatmap. MIA–MIA and AIS–IAC both have score 2 and are shown as separate, tied categories (dashed lines). Values within cells indicate percentage positivity, with positive/evaluable case counts shown in parentheses (e.g., 77.8% [21/27]). The color scale spans the full 0–100% range. Abbreviations: AIS, adenocarcinoma in situ; MIA, minimally invasive adenocarcinoma; IAC, invasive adenocarcinoma; PD-L1, programmed death-ligand 1; TPS, tumor proportion score; STAS, spread through air spaces.

To characterize the relationship between paired pathological invasiveness and tumor aggressiveness, we evaluated six Lesion 1 aggressiveness markers across the six pathological combinations, ordered by the pre-specified WHO-based paired invasiveness score (AIS = 0, MIA = 1, IAC = 2; total range 0–4; Figure 2). This ordering was defined independently of the aggressiveness markers under evaluation. MIA–MIA and AIS–IAC both scored 2 and were displayed separately without imposing an unsupported order between them. Because concordance is defined by matching grade rather than matching risk, the match group spans both low-risk (AIS–AIS) and high-risk (IAC–IAC) pairings. The adverse prognostic signal reported in Section 3.4 pertains specifically to the IAC + IAC pairing, which represents the highest paired invasiveness score.

IAC–IAC combinations (*n* = 34) showed the highest positivity for most markers, including PD-L1 TPS ≥ 1% (77.8%, 21/27), Ki-67 ≥ 10% (59.3%, 16/27), and STAS (23.5%, 8/34). STAS was concentrated in IAC-containing combinations, occurring in 8/34 IAC–IAC and 3/40 MIA–IAC pairs, and in none of the remaining four combinations.

Cochran–Armitage tests showed significant positive linear trends across the paired invasiveness score for PD-L1 TPS ≥ 1%, Ki-67 ≥ 10%, Ki-67 ≥ 20%, STAS, and pleural invasion (all *p* ≤ 0.020; Supplementary Table S1). Vascular invasion showed a positive but non-significant trend (*p* = 0.113), consistent with its low overall frequency (2 positive cases). PD-L1 and Ki-67 analyses were limited to patients with available immunohistochemistry (*n* = 92 and *n* = 97, respectively). These findings extend beyond the binary match/mismatch classification, identifying IAC–IAC as a candidate biologically aggressive subgroup within the broader match category.

### 3.4. Prognostic Impact of the Second Lesion’s Invasive Subtype (IAC vs. AIS/MIA) in the IAC-Dominant Subset

Among 91 patients with IAC as the dominant lesion, 34 were classified as IAC + IAC and 57 as IAC + AIS/MIA. Demographic characteristics (age, sex, smoking history) were comparable between groups (all *p* > 0.05). The overall pathological stage and Lesion 1 T stage did not differ significantly between groups (*p* = 0.186 and *p* = 0.085, respectively), whereas Lesion 2 T stage did (*p* < 0.001). Lesion 1 aggressiveness markers were significantly higher in the IAC + IAC group, including STAS positivity (23.5% vs. 5.3%, *p* = 0.017), Ki-67 ≥ 10% (59.3% vs. 34.1%, *p* = 0.050), and PD-L1 TPS ≥ 1% (77.8% vs. 32.6%, *p* < 0.001). Same-lobe location was also markedly less frequent in the IAC + IAC group (8.8% vs. 68.4%, *p* < 0.001), mirroring its association with the pathological mismatch identified in the full cohort (Section 3.2). The surgical procedure for the dominant lesion was comparable between groups (76.5% vs. 70.2% lobectomy-dominant, *p* = 0.630), although the detailed four-category distribution differed (*p* < 0.001), primarily reflecting more lobectomy plus sublobar resections in the IAC + IAC group (67.6% vs. 15.8%), consistent with their predominantly different-lobe anatomy (Supplementary Table S2).

**Figure 3:**
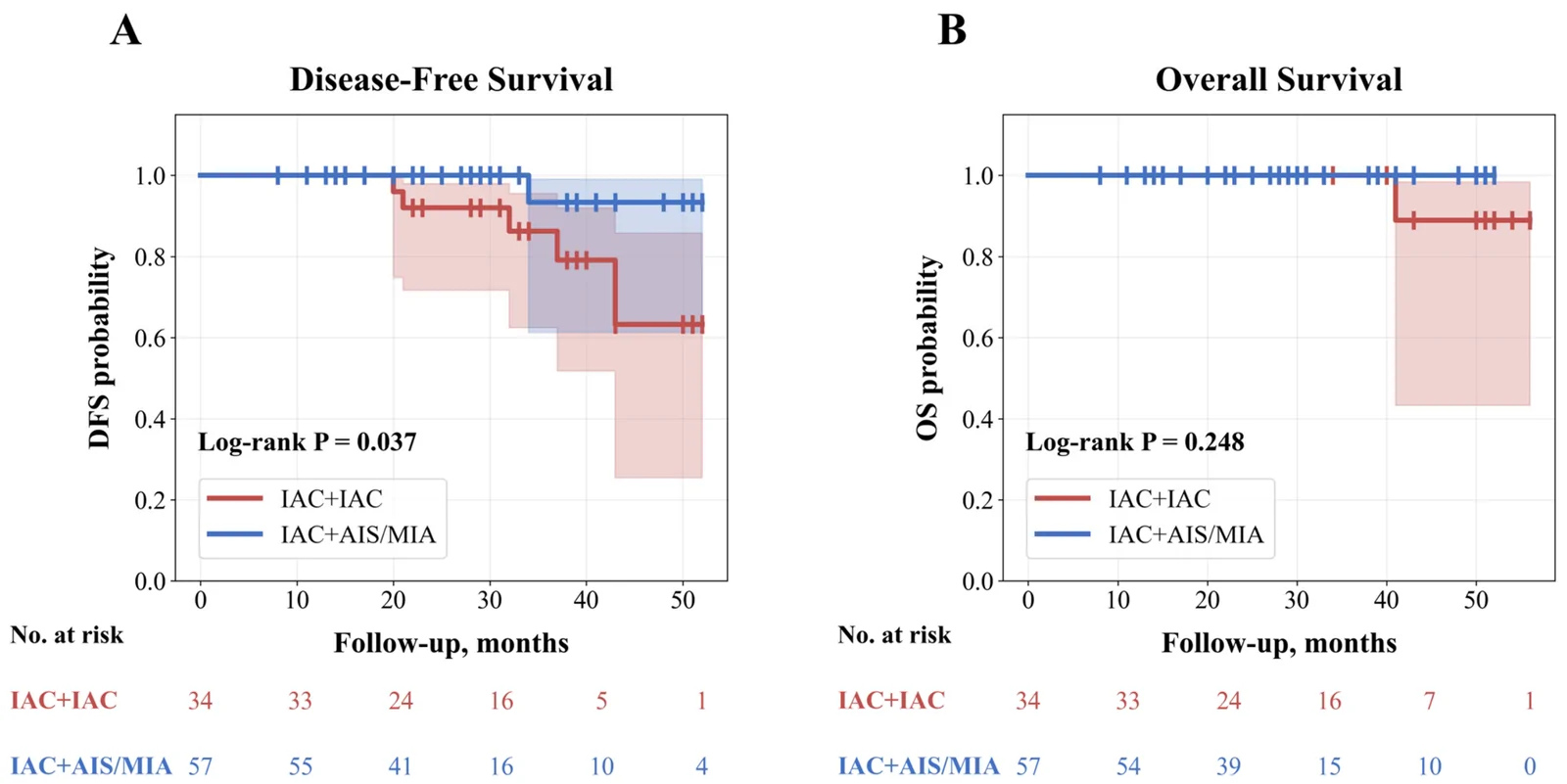
Kaplan–Meier curves for (**A**) DFS and (**B**) OS in the IAC-dominant subset (*n* = 91). Abbreviations: DFS, disease-free survival; HR, hazard ratio; IAC, invasive adenocarcinoma; MIA, minimally invasive adenocarcinoma; AIS, adenocarcinoma in situ; OS, overall survival.

The 34 IAC + IAC cases comprised three same-lobe pairs (with NGS-confirmed clonal independence) and 31 different-lobe pairs (classified by ACCP criteria). The median follow-up was 29.0 months (IQR 17.0–39.0; range 8.0–56.0) for the overall IAC-dominant subset. Follow-up durations were comparable between groups (Mann–Whitney U test, *p* = 0.171): 33.0 months (IQR 18.0–41.0; range 8.0–56.0) in IAC + IAC and 28.0 months (IQR 17.0–38.0; range 8.0–52.0) in IAC + AIS/MIA. Given the small number of DFS events (6 total; 5 vs. 1), this survival analysis was strictly exploratory and should be interpreted with caution. Six DFS events occurred: 5/34 (14.7%) in the IAC + IAC group (2 visceral metastases, 2 pulmonary recurrences, 1 brain metastasis) versus 1/57 (1.8%) in the IAC + AIS/MIA group (1 visceral metastasis). The surgical procedures in these six patients were as follows: three underwent lobectomy plus sublobar resection, two underwent lobectomy alone, and one underwent sublobar plus sublobar resection. Kaplan–Meier analysis showed significantly worse DFS in the IAC + IAC group (log-rank *p* = 0.037, Figure 3A). Univariate Firth-penalized Cox regression showed that IAC-IAC concordance was associated with an increased recurrence risk relative to IAC + AIS/MIA (HR = 5.28, 95% CI 1.04–51.84, *p* = 0.044; Table 3).

Two sensitivity analyses assessed potential confounding by Lesion 1 tumor burden, each adding a single covariate given the severe event-per-variable constraint (6 events). Adjustment for Lesion 1 T stage yielded HR = 4.66 (95% CI 0.84–46.91, *p* = 0.079); adjustment for Lesion 1 size yielded HR = 4.03 (95% CI 0.73–40.85, *p* = 0.114). The direction of association remained consistent across all models despite attenuated, non-significant estimates after adjustment (Table 3), and this pattern should therefore be interpreted as preliminary rather than confirmatory. A further potential confounder is the marked same-lobe imbalance between groups (8.8% vs. 68.4%, *p* < 0.001). Given that same-lobe location was independently associated with pathological mismatch in the full cohort (adjusted OR = 2.26, *p* = 0.017), this anatomical difference may also contribute to the observed DFS disparity. However, formal adjustment was not feasible with only six events, and residual confounding cannot be excluded.

The overall survival could not be formally modeled, as only one death occurred during follow-up, in the IAC + IAC group (Figure 3B, descriptive only). This death was attributed to a cerebrovascular event during the treatment period, likely related to therapy rather than disease progression.

## 4. Discussion

This study examined inter-lesion pathological relationships in SMPLA at three levels: a preoperative predictive model for histological concordance between paired lesions, the internal consistency of a clinicopathological aggressiveness gradient across paired lesions, and the prognostic significance of pairing status within an IAC-dominant subgroup.

### 4.1. Preoperative Prediction of Inter-Lesion Concordance: Value and Ceiling

Our three-variable model (same-lobe location, Lesion 1 size, Lesion 2 size) achieved an AUC of 0.671 for predicting histological concordance between paired lesions. On its own, this discriminative performance looks modest, but it sits comfortably within the range reported by comparable preoperative or clinicopathological prediction models in MPLC populations: SEER-based nomograms (Luo et al., AUC 0.70–0.76 across staging comparisons) [21], a multicenter survival-prediction model for synchronous MPLC (Ji et al.) [22], and a SEER-based nomogram for double primary lung cancer (Song et al.) [23] all report AUCs in the 0.65–0.78 range when relying on clinical, radiological, or staging variables alone. This convergence across independent cohorts and modeling strategies suggests that the ceiling we observed is a genuine biological limitation rather than a shortcoming of our variable selection. Whether two synchronous lesions share the same invasive subtype is, at its core, a histopathological and probably a molecular question, and anatomical or radiological surrogates such as location and size can only partly stand in for it. We therefore position our model as a preoperative risk-stratification aid for planning biopsy and surveillance intensity, rather than as a tool for clinical decision-making. External validation in independent cohorts will be necessary before this model can be considered for routine clinical use.

### 4.2. Clinicopathological Aggressiveness Gradient Across Paired Lesions

The prognostic relevance of AIS, MIA, and IAC subtyping is well established at the single-lesion level. STAS, first characterized as an invasive growth pattern predictive of recurrence, has since been validated across several independent stage I adenocarcinoma cohorts. Shiono and Yanagawa followed 318 patients with stage I lung adenocarcinoma and found that STAS positivity was associated with markedly worse 5-year overall survival (62.7% vs. 91.1%) and recurrence-free survival (54.4% vs. 87.8%), with STAS remaining an independent prognostic factor on multivariate analysis [24]. A later meta-analysis pooling 3564 patients across 12 studies confirmed STAS as a consistent adverse prognostic factor for both recurrence-free and overall survival in non-small cell lung cancer [25]. Our Cochran–Armitage trend analysis extends this single-lesion evidence to the paired-lesion setting: PD-L1 TPS ≥ 1%, Ki-67 ≥ 10%, and Ki-67 ≥ 20% positivity, STAS, and pleural invasion all showed statistically significant monotonic increases across the AIS → MIA → IAC gradient (all *p* ≤ 0.02), while vascular invasion did not reach significance in our cohort. This indicates that the WHO/IASLC invasiveness classification—defined purely on morphological grounds—captures a coherent clinicopathological aggressiveness gradient that reproduces across paired lesions within the same patient, supporting its use as a practical proxy for biological aggressiveness in the multi-lesion setting. The PD-L1 and Ki-67 trend analyses were based on subsets with non-randomly missing immunohistochemistry and should therefore be interpreted cautiously.

### 4.3. Inter-Lesion Concordance and Prognosis: A Convergent but Heterogeneous Evidence Base

To contextualize our findings, we identified three independent MPLC cohorts that stratified patients by inter-lesion concordance or discordance, rather than by single-lesion features alone, and reported a quantifiable survival or recurrence outcome (Table 4). Despite differing concordance definitions, follow-up durations, and statistical approaches, a directionally consistent theme emerged: the pathological relationship between paired lesions—not either lesion analyzed alone—carried prognostic information. In our IAC-dominant subset, IAC + IAC pairing was associated with elevated recurrence risk (HR = 5.28; 5 recurrences among 34 IAC + IAC patients versus 1 among 57 IAC + AIS/MIA patients), directionally consistent with Qiu et al.’s bi-STAS cohort, where both lesions sharing STAS positivity independently predicted worse recurrence-free survival (HR = 3.99) [11]. Notably, this recurrence disparity was not attributable to overall disease burden: overall stage and Lesion 1 T stage were comparable between groups, and only Lesion 2 T stage differed significantly.

This recurrence disparity, while directionally consistent with prior data, derives from only six events with severe asymmetry (5 vs. 1), producing a wide confidence interval (1.04–51.84). A distinguishing feature of the prognostic analysis is that it was restricted to the IAC-dominant subgroup: because Lesion 1 was uniformly IAC in both groups, the observed DFS difference depends exclusively on Lesion 2 histology and cannot be attributed to structural dependencies that are inherent in the Lesion 1 definition. These findings should therefore be interpreted as preliminary and hypothesis-generating rather than confirmatory, pending independent external validation. Full-cohort descriptive analyses, including the clinicopathological aggressiveness gradient, should be understood as characterizations of how concordance distributes across the cohort rather than as independent biological findings.

Among the 34 IAC + IAC pairs, molecular confirmation of clonal independence was available only for the three same-lobe cases; the remaining 31 different-lobe pairs were classified by ACCP criteria without molecular testing. Since this latter group contributed the majority of DFS events, the possibility that some of these 31 pairs represent occult intrapulmonary metastases rather than true independent primaries cannot be entirely excluded, though standard clinicopathological criteria suggest this is unlikely. The marked same-lobe imbalance between groups (8.8% vs. 68.4%) may also act as an unmeasured confounder, given its independent association with pathological mismatch in the full cohort.

Yang et al., in a related cohort of 72 postoperative synchronous MPLC patients, found that the presence of any pathological high-risk factor, rather than specific driver-gene status (EGFR/TP53/KRAS), predicted DFS (*p* < 0.001) [26], echoing our own observation that immunohistochemical markers of proliferative and immune activity (Ki-67, PD-L1), rather than molecular profiling, tracked with pathological concordance in our cohort. A fourth cohort, Zhang et al., also stratified patients by pathological concordance and reported worse overall survival in discordant pairs (HR = 9.73) [27], but their definition of concordance compared histological type rather than invasiveness grade, and is therefore not directly comparable to our framework or Qiu et al.’s. Event numbers across all four studies were small (ranging from 5 to 34 outcome events depending on the stratum), and we regard this signal as hypothesis-generating pending validation in larger, prospectively designed cohorts with a harmonized concordance definition.

Existing evidence on STAS specifically in the MPLC setting has largely been generated at the patient level rather than the paired-lesion level. Xie et al. studied 122 stage IA–IB MPLC patients and defined STAS positivity at the patient level—present if detected in at least one lesion—reporting it as an independent predictor of recurrence alongside tumor diameter and pleural invasion [28]. Because STAS status was not cross-tabulated between the two lesions within each patient, this patient-level framework cannot distinguish whether recurrence risk is driven by STAS burden in a single dominant lesion or by concordant STAS positivity across both lesions—a distinction that our paired-lesion design and Qiu et al.’s bi-STAS/mono-STAS/un-STAS stratification were built specifically to resolve. Together with the comparisons in Table 4, this observation points to a broader pattern in the field: most existing MPLC prognostic studies characterize lesions individually or aggregate risk at the patient level, and relatively few adopt an explicitly paired, inter-lesion analytic framework. We regard this paired-lesion framework, rather than any single biomarker finding, as the primary methodological contribution of the present study.

The same principle applies at the immunophenotypic level. Mansfield et al. performed PD-L1 immunohistochemistry on paired lesions from 79 patients with multifocal lung adenocarcinoma and found that independent primary tumors showed poor inter-lesion PD-L1 concordance (κ = 0.31), in contrast to high concordance between lesions confirmed as intrapulmonary metastases (κ = 0.73) [29]. Our finding that PD-L1 expression differed systematically between paired lesions—with Lesion 1 showing higher TPS than Lesion 2 in the match group—fits this framework and reinforces, at the level of an actionable immune biomarker, the same point illustrated above by Xie et al. and Qiu et al.: assessment restricted to a single lesion or to the patient level can obscure clinically meaningful inter-lesion heterogeneity. Our data support obtaining PD-L1 assessment from more than one lesion, rather than relying on a single dominant-lesion biopsy, when immunotherapy eligibility is being considered in patients with synchronous multifocal disease, since a single biopsy site risks misclassifying a patient’s true multi-lesion PD-L1 status—although whether this practice changes treatment outcomes has not been tested in prospective trials.

**Table 4:** Comparison of prognostic stratification in multiple primary lung adenocarcinoma cohorts.

Study	*N*	Design	Follow-Up (mo)	Stratification	High-Risk Group (*n*)	Outcome (DFS/RFS/OS)	*p* Value	Key Prognostic Biomarker
Present Study	91 (IAC subset)	Retrospective cohort	30	IAC + IAC vs. IAC + AIS/MIA	34	DFS: 5/34 (14.7%) vs. 1/57 (1.8%)	0.044 (univariate Firth-Cox)	Ki67 ≥ 10%: 59.3% vs. 34.1% (*p* = 0.050); PD-L1 TPS ≥ 1%: 77.8% vs. 32.6% (*p* = 0.001)
Qiu et al. (2025) [11]	131	Retrospective cohort	81	Bi-STAS vs. Mono-STAS vs. un-STAS	21 (Bi-STAS)	RFS HR = 3.99 (95% CI 1.34–11.93)	<0.001	Bi-STAS independent predictor of both RFS (*p* = 0.013) and OS (HR = 3.78, *p* = 0.020)
Zhang et al. (2024) [27]	293	Retrospective cohort	62	Identical vs. different histological type	8 (different)	5-yr OS: 96.7% vs. 75.0%	0.001	Different histological type independent predictor of worse OS (HR = 9.726, 95% CI 1.886–50.151)
Yang et al. (2025) [26]	72	Retrospective cohort	24	Pathological high-risk factor vs. none (stage IA subset)	5 (stage IA subset with ≥1 pathological high-risk factor)	DFS: 24 mo vs. Not reached	<0.001	PD-L1 ≥ 1% positive: *p* = 0.008; EGFR/TP53/KRAS mutation status: not significant

Note: DFS, disease-free survival; RFS, recurrence-free survival; OS, overall survival; STAS, spread through air spaces; Bi-STAS, both tumors STAS-positive; Mono-STAS, one tumor STAS-positive; Un-STAS, both tumors STAS-negative (Qiu et al., 2025) [11]. HR, hazard ratio; CI, confidence interval.

### 4.4. Limitations

Several limitations should be considered when interpreting these findings. First, recurrence events in the IAC-dominant subgroup were sparse (5/34 IAC + IAC vs. 1/57 IAC + AIS/MIA), producing wide confidence intervals around the HR for the IAC + IAC versus IAC + AIS/MIA comparison. Although Firth-penalized Cox regression was used to mitigate small-sample bias [30], given the limited event count, this analysis should still be regarded as exploratory rather than confirmatory. Second, this was a single-center, retrospective study. The cohort reflects the patient population and clinical practices of a specialized thoracic surgery center, and the findings may not be directly applicable to other institutions or healthcare systems with different approaches to MPLC management. The retrospective design also leaves open the possibility of unmeasured confounding in lesion selection or surgical decision-making. Prospective multicenter validation will therefore be necessary before the paired-lesion framework can be considered for broader clinical application. Third, molecular confirmation of clonal independence was not uniformly performed. This distinction is only biologically relevant for IAC–IAC pairs, since lesion pairs with lower-grade components cannot represent metastatic disease. Among IAC–IAC pairs, 31/34 were in different lobes and were classified according to ACCP criteria without molecular testing; this is consistent with standard clinical practice, but occult intrapulmonary metastasis cannot be entirely excluded in this subgroup, which contributed the majority of DFS events. The remaining same-lobe IAC–IAC pairs (*n* = 3) underwent next-generation sequencing, which confirmed independent clonal origin in each instance. Fourth, several analytic limitations warrant cautious interpretation of the reported *p*-values. Ki-67 and PD-L1 immunohistochemistry were not routinely performed on AIS or MIA lesions, resulting in missing data for approximately half of the cohort and potential selection bias, as testing was preferentially ordered for larger or clinically more suspicious lesions. The clinicopathological gradient findings involving these two markers should therefore be viewed as supportive rather than definitive. Additionally, multiple statistical comparisons were conducted across the study—including 34 candidate variables screened univariably in the preoperative model and six aggressiveness markers tested for trend—without formal multiplicity correction. The *p*-values should therefore be interpreted as describing a broadly coherent pattern rather than as independently confirmed hypotheses. Finally, the follow-up was adequate to capture early recurrence but possibly too short to fully characterize the natural history of indolent AIS/MIA-containing lesion pairs, some of which may recur or progress only after several years.

## 5. Conclusions

In this cohort of SMPLA patients, preoperative anatomical and size variables provided modest but statistically genuine discrimination of inter-lesion histological concordance, and the WHO/IASLC invasiveness gradient tracked coherently with most independent clinicopathological aggressiveness markers across paired lesions. Within the IAC-dominant subgroup, the IAC + IAC pairing showed a preliminary, statistically fragile association with higher recurrence risk (6 events; HR = 5.28, 95% CI 1.04–51.84, *p* = 0.044), which should be interpreted as hypothesis-generating rather than confirmatory. This unfavorable recurrence signal was specific to the IAC + IAC pairing and may not apply to other concordant configurations. Together, these findings suggest that a paired-lesion analytic framework may complement conventional single-lesion characterization in MPLC. Future work should prioritize validating this framework in prospective, molecularly annotated cohorts with standardized concordance definitions and longer follow-up, to determine whether inter-lesion pathological relationships provide independent prognostic value and warrant incorporation into risk-stratification models for SMPLA.

## Figures and Tables

**Table 1:** Univariate and multivariable logistic regression for predictors of pathological mismatch.

Variable	Univariate OR (95% CI)	*p* (Univariate)	Multivariable OR (95% CI)	*p* (Multivariate)
Lesion 1 T Stage–Tis	0.249 (0.111–0.558)	**<0.001**	—	—
Lesion 1 T Stage–T1b	3.421 (1.626–7.197)	**0.001**	—	—
Lesion 1 PD-L1 TPS ≥ 1% ^a^	0.300 (0.126–0.714)	**0.006**	—	—
Lesion 2 T Stage–Tis	2.163 (1.207–3.875)	**0.010**	—	—
Same lobe	2.176 (1.181–4.009)	**0.013**	2.260 (1.158–4.410)	**0.017**
Lesion 1 location–RLL	3.040 (1.262–7.326)	**0.013**	—	—
Lesion 2 size, mm	0.896 (0.814–0.987)	**0.026**	0.786 (0.685–0.902)	**<0.001**
Lesion 2 T Stage–T1mi	1.815 (1.030–3.199)	**0.039**	—	—
Lesion 1 PD-L1 TPS, %	0.904 (0.808–1.011)	0.078	—	—
Lesion 1 T Stage–T1a	1.892 (0.882–4.056)	0.101	—	—
Lesion 1 T Stage–T2a	0.213 (0.024–1.856)	0.162	—	—
Lesion 1 STAS positive	0.396 (0.102–1.538)	0.181	—	—
Lesion 1 location–RML	0.475 (0.159–1.422)	0.183	—	—
Lesion 1 size, mm	1.029 (0.983–1.078)	0.221	1.137 (1.058–1.221)	**<0.001**
Lesion 1 pleural invasion	0.269 (0.030–2.449)	0.244	—	—
Lesion 1 T Stage–T1mi	0.707 (0.390–1.281)	0.253	—	—
Lesion 1 T Stage–T1c	2.011 (0.570–7.103)	0.278	—	—
Lesion 1 location–LUL	0.714 (0.387–1.317)	0.281	—	—
Lesion count	1.300 (0.781–2.165)	0.313	—	—
Lesion 2 location–LUL	0.771 (0.414–1.439)	0.414	—	—
Lesion 1 location–LLL	1.230 (0.531–2.849)	0.628	—	—
Lesion 2 location–RML	1.271 (0.469–3.440)	0.637	—	—
Lesion 2 location–RUL	1.121 (0.626–2.004)	0.701	—	—
Lesion 1 location–RUL	0.896 (0.500–1.608)	0.714	—	—
Age, years ^b^	1.004 (0.979–1.030)	0.748	—	—
Gender (male) ^b^	1.101 (0.598–2.024)	0.758	—	—
Smoking history	1.078 (0.606–1.918)	0.798	—	—
LN N1 positive	1.110 (0.219–5.638)	0.900	—	—
Lesion 2 location–LLL	1.044 (0.485–2.249)	0.912	—	—
Lesion 1 vascular invasion	1.108 (0.068–17.959)	0.943	—	—
Lesion 2 location–RLL	1.016 (0.426–2.426)	0.971	—	—
Lesion 2 T Stage–T1a	0.000 (0.000–inf)	0.999	—	—
Lesion 2 T Stage–T1b	0.000 (0.000–inf)	1.000	—	—
Lesion 2 T Stage–T1c	0.000 (0.000–inf)	1.000	—	—

Abbreviations: OR, odds ratio; CI, confidence interval. Bold *p*-values indicate *p* < 0.05. The multivariable model is the parsimonious preoperative model (same-lobe location, Lesion 1 size, Lesion 2 size), fitted on all patients with complete preoperative data (*N* = 198); the Lesion 1 T stage and Lesion 1 location were excluded a priori from the multivariable model owing to definitional overlap with the mismatch outcome and multiplicity across lobe categories, respectively, and are shown only in univariate form. For T-stage and lobe-location categories, each univariable OR represents that category versus all remaining categories within the same variable (one-vs-rest coding), not versus a single reference category. — denotes variables not included in the multivariable model. ^a^ Lesion 1 PD-L1 TPS ≥ 1% was evaluated separately in a sensitivity analysis restricted to the 92 patients with available PD-L1 data (see text); it was not included in the primary multivariable model. ^b^ Age and sex were tested in an extended 5-variable model (age OR = 1.008, *p* = 0.582; sex OR = 0.929, *p* = 0.830) but contributed no discriminative information, individually or jointly (likelihood-ratio test, LR = 0.32, df = 2, *p* = 0.852), and were therefore excluded from the final parsimonious multivariable model shown here.

**Table 2:** Baseline characteristics of the study cohort stratified by pathological match status.

Variable	Total (*N* = 198)	Match (*N* = 104)	Mismatch (*N* = 94)	*p*
**Demographics**				
Age, years	57 [50–65]	57 [50–64]	58 [50–66]	0.735
**Gender**				0.876
Female	139/198 (70.2%)	74/104 (71.2%)	65/94 (69.1%)	
Male	59/198 (29.8%)	30/104 (28.8%)	29/94 (30.9%)	
**Smoking history**				0.883
Yes	74/198 (37.4%)	38/104 (36.5%)	36/94 (38.3%)	
No	124/198 (62.6%)	66/104 (63.5%)	58/94 (61.7%)	
**Tumor characteristics**				
Lesion 1 size, mm	9 [6–12]	8 [6–11]	10 [7–14]	0.059
Lesion 2 size, mm	5 [4–7]	5 [4–7]	5 [4–7]	0.085
**Surgical procedure (dominant lesion)**				0.658
Lobectomy (dominant)	72/198 (36.4%)	36/104 (34.6%)	36/94 (38.3%)	
Sublobar (dominant)	126/198 (63.6%)	68/104 (65.4%)	58/94 (61.7%)	
**Detailed procedure**				**0.002**
Lobectomy	38/198 (19.2%)	11/104 (10.6%)	27/94 (28.7%)	
Sublobar resection	94/198 (47.5%)	50/104 (48.1%)	44/94 (46.8%)	
Lobectomy + sublobar	34/198 (17.2%)	25/104 (24.0%)	9/94 (9.6%)	
Sublobar + sublobar	32/198 (16.2%)	18/104 (17.3%)	14/94 (14.9%)	
LN N1 positive, *n*/*N* (%)	6/198 (3.0%)	3/104 (2.9%)	3/94 (3.2%)	1.000
Lesion count	2 [2–2]	2 [2–2]	2 [2–2]	0.574
**Same lobe**				**0.016**
Yes	132/198 (66.7%)	61/104 (58.7%)	71/94 (75.5%)	
No	66/198 (33.3%)	43/104 (41.3%)	23/94 (24.5%)	
**Overall clinical stage**				**<0.001**
Stage 0	40/198 (20.2%)	31/104 (29.8%)	9/94 (9.6%)	
Stage IA1	99/198 (50.0%)	52/104 (50.0%)	47/94 (50.0%)	
Stage IA2	41/198 (20.7%)	12/104 (11.5%)	29/94 (30.9%)	
Stage IA3	9/198 (4.5%)	3/104 (2.9%)	6/94 (6.4%)	
Stage IB	3/198 (1.5%)	3/104 (2.9%)	0/94 (0.0%)	
Stage IIA	3/198 (1.5%)	1/104 (1.0%)	2/94 (2.1%)	
Stage IIB	3/198 (1.5%)	2/104 (1.9%)	1/94 (1.1%)	
**Lesion 1 T stage**				**<0.001**
Tis	40/198 (20.2%)	31/104 (29.8%)	9/94 (9.6%)	
T1mi	67/198 (33.8%)	39/104 (37.5%)	28/94 (29.8%)	
T1a	33/198 (16.7%)	13/104 (12.5%)	20/94 (21.3%)	
T1b	41/198 (20.7%)	12/104 (11.5%)	29/94 (30.9%)	
T1c	11/198 (5.6%)	4/104 (3.8%)	7/94 (7.4%)	
T2a	6/198 (3.0%)	5/104 (4.8%)	1/94 (1.1%)	
**Lesion 2 T stage**				**<0.001**
Tis	76/198 (38.4%)	31/104 (29.8%)	45/94 (47.9%)	
T1mi	88/198 (44.4%)	39/104 (37.5%)	49/94 (52.1%)	
T1a	24/198 (12.1%)	24/104 (23.1%)	0/94 (0.0%)	
T1b	9/198 (4.5%)	9/104 (8.7%)	0/94 (0.0%)	
T1c	1/198 (0.5%)	1/104 (1.0%)	0/94 (0.0%)	
**Lesion 1 location**				0.076
Right upper lobe	70/198 (35.4%)	38/104 (36.5%)	32/94 (34.0%)	
Right middle lobe	16/198 (8.1%)	11/104 (10.6%)	5/94 (5.3%)	
Right lower lobe	27/198 (13.6%)	8/104 (7.7%)	19/94 (20.2%)	
Left upper lobe	60/198 (30.3%)	35/104 (33.7%)	25/94 (26.6%)	
Left lower lobe	25/198 (12.6%)	12/104 (11.5%)	13/94 (13.8%)	
**Lesion 2 location**				0.940
Right upper lobe	71/198 (35.9%)	36/104 (34.6%)	35/94 (37.2%)	
Right middle lobe	17/198 (8.6%)	8/104 (7.7%)	9/94 (9.6%)	
Right lower lobe	23/198 (11.6%)	12/104 (11.5%)	11/94 (11.7%)	
Left upper lobe	56/198 (28.3%)	32/104 (30.8%)	24/94 (25.5%)	
Left lower lobe	31/198 (15.7%)	16/104 (15.4%)	15/94 (16.0%)	
**Pathology**				
**Lesion 1 pathology**				**<0.001**
IAC	91/198 (46.0%)	34/104 (32.7%)	57/94 (60.6%)	
MIA	67/198 (33.8%)	39/104 (37.5%)	28/94 (29.8%)	
AIS	40/198 (20.2%)	31/104 (29.8%)	9/94 (9.6%)	
**Lesion 2 pathology**				**<0.001**
IAC	34/198 (17.2%)	34/104 (32.7%)	0/94 (0.0%)	
MIA	88/198 (44.4%)	39/104 (37.5%)	49/94 (52.1%)	
AIS	76/198 (38.4%)	31/104 (29.8%)	45/94 (47.9%)	
**Dominant histological subtype**				
Lesion 1 dominant subtype (*n* = 82 invasive) *				0.645
Acinar	70/82 (85.4%)	29/33 (87.9%)	41/49 (83.7%)	
Papillary	2/82 (2.4%)	1/33 (3.0%)	1/49 (2.0%)	
Lepidic	6/82 (7.3%)	1/33 (3.0%)	5/49 (10.2%)	
Complex glandular	4/82 (4.9%)	2/33 (6.1%)	2/49 (4.1%)	
Lesion 2 dominant subtype (*n* = 32 invasive) *				N/A
Acinar	24/32 (75.0%)	24/32 (75.0%)	N/A	
Papillary	3/32 (9.4%)	3/32 (9.4%)	N/A	
Lepidic	5/32 (15.6%)	5/32 (15.6%)	N/A	
**Immunohistochemistry (Lesion 1)**				
Ki67, % (*n* = 97)	5.0 [3.0–10.0]	5.0 [3.0–10.0]	5.0 [3.0–10.0]	0.901
PD-L1 TPS, % (*n* = 92)	0.8 [0.0–1.0]	1.0 [0.8–3.5]	0.8 [0.0–1.0]	**0.005**

Continuous variables are summarized as median [interquartile range] and compared using the Mann–Whitney U test because most subgroups showed non-normal distribution (Shapiro–Wilk test, *p* < 0.05 for all except age in the match group). Surgical procedures were categorized according to the extent of resection for the dominant lesion (Lesion 1). “Lobectomy (dominant)” includes lobectomy alone and lobectomy plus sublobar resection for the secondary lesion; “Sublobar (dominant)” includes sublobar resection alone and sublobar resection for both lesions. Detailed four-category classification is also shown. * Dominant subtype percentages are calculated among lesions with a recorded invasive pattern; cases without an invasive pattern (true zero) and those with unrecorded subtype breakdowns are excluded from the denominator and *p* value calculation. For the Lesion 2 dominant subtype, no invasive pattern was recorded in the mismatch group, yielding an undefined *p* value (N/A).

**Table 3:** Disease-free survival according to Lesion 2 pathological concordance in the IAC-dominant subset.

Analysis	Events, *n*	HR	95% CI	*p*
**Primary analysis**				
Log-rank test	6/91	—	—	**0.037**
Univariate Firth-penalized Cox	6/91	5.28	1.04–51.84	**0.044**
**Sensitivity analyses (Firth-penalized Cox, adjusted)**				
+Lesion 1 T stage (ordinal)	6/91	4.66	0.84–46.91	0.079
+Lesion 1 size (continuous, mm)	6/91	4.03	0.73–40.85	0.114
**Event distribution by group**				
IAC + IAC	5/34 (14.7%)	—	—	—
IAC + AIS/MIA (reference)	1/57 (1.8%)	—	—	—
**Overall survival**				
Descriptive only (1 total event)	1/91	not modeled	—	—

HR estimated by Firth’s penalized-likelihood Cox regression (profile-likelihood CI) to address the rare-event setting (*n* = 6 DFS events). Abbreviations: CI, confidence interval; DFS, disease-free survival; HR, hazard ratio; IAC, invasive adenocarcinoma; MIA, minimally invasive adenocarcinoma; AIS, adenocarcinoma in situ; OS, overall survival; EPV, events per variable.

## Data Availability

The data presented in this study are available upon request from the corresponding author.

## Supplementary Materials

The following supporting information can be downloaded at https://www.mdpi.com/article/10.3390/jcm15187317/s1, Table S1: Cochran–Armitage trend tests for Lesion 1 aggressiveness markers across the WHO-defined paired invasiveness score; Table S2: Baseline characteristics of the IAC-dominant subset (*n* = 91).

## References

[1] Chen C., Huang X., Peng M., Liu W., Yu F., Wang X. (2019). Multiple primary lung cancer: A rising challenge. J. Thorac. Dis..

[2] Samadzadeh Tabrizi N., Gallant B., Harris E., Arnold B., Fabian T. (2023). Contemporary Incidence of Synchronous Multiple Primary Lung Cancers and Survival in the Era of Lung Cancer Screening. Innov. Technol. Tech. Cardiothorac. Vasc. Surg..

[3] Tian H., Bai G., Yang Z., Chen P., Xu J., Liu T., Fan T., Wang B., Xiao C., Li C. (2023). Multiple primary lung cancer: Updates of clinical management and genomic features. Front. Oncol..

[4] Guo H., Mao F., Zhang H., Qiu Y., Shen T. (2017). Analysis on the Prognostic and Survival Factors of Synchronous Multiple Primary Lung Cancer. Chin. J. Lung. Cancer.

[5] Jiang L., He J., Shi X., Shen J., Liang W., Yang C., He J. (2015). Prognosis of synchronous and metachronous multiple primary lung cancers: Systematic review and meta-analysis. Lung Cancer.

[6] Detterbeck F.C., Franklin W.A., Nicholson A.G., Girard N., Arenberg D.A., Travis W.D., Mazzone P.J., Marom E.M., Donington J.S., Tanoue L.T. (2016). The IASLC Lung Cancer Staging Project: Background Data and Proposed Criteria to Distinguish Separate Primary Lung Cancers from Metastatic Foci in Patients with Two Lung Tumors in the Forthcoming Eighth Edition of the TNM Classification for Lung Cancer. J. Thorac. Oncol..

[7] Qu R., Ye F., Tu D., Cai Y., Fu X. (2021). Clinical Features and Surgical Treatment of Synchronous Multiple Primary Lung Adenocarcinomas With Different EGFR Mutations. Front. Oncol..

[8] Wu C., Zhao C., Yang Y., He Y., Hou L., Li X., Gao G., Shi J., Ren S., Chu H. (2015). High Discrepancy of Driver Mutations in Patients with NSCLC and Synchronous Multiple Lung Ground-Glass Nodules. J. Thorac. Oncol..

[9] Yang Z., Zhou B., Guo W., Peng Y., Tian H., Xu J., Wang S., Chen X., Hu B., Liu C. (2024). Genomic characteristics and immune landscape of super multiple primary lung cancer. eBioMedicine.

[10] Travis W.D., Brambilla E., Noguchi M., Nicholson A.G., Geisinger K.R., Yatabe Y., Beer D.G., Powell C.A., Riely G.J., Van Schil P.E. (2011). International Association for the Study of Lung Cancer/American Thoracic Society/European Respiratory Society International Multidisciplinary Classification of Lung Adenocarcinoma. J. Thorac. Oncol..

[11] Qiu T., Hou F., Wu J., Wu Z., Du W., Yang J., Zhao Y., Jin X., Wang Z., Tian K. (2025). Concurrent spread through air spaces in dominant tumours impacts prognosis in synchronous multiple primary lung adenocarcinoma. Eur. J. Cardiothorac. Surg..

[12] Jakobsen J.N., Sørensen J.B. (2013). Clinical impact of ki-67 labeling index in non-small cell lung cancer. Lung Cancer.

[13] Roach C., Zhang N., Corigliano E., Jansson M., Toland G., Ponto G., Dolled-Filhart M., Emancipator K., Stanforth D., Kulangara K. (2016). Development of a Companion Diagnostic PD-L1 Immunohistochemistry Assay for Pembrolizumab Therapy in Non-Small-cell Lung Cancer. Appl. Immunohistochem. Mol. Morphol..

[14] Kadota K., Nitadori J.I., Sima C.S., Ujiie H., Rizk N.P., Jones D.R., Adusumilli P.S., Travis W.D. (2015). Tumor Spread through Air Spaces is an Important Pattern of Invasion and Impacts the Frequency and Location of Recurrences after Limited Resection for Small Stage I Lung Adenocarcinomas. J. Thorac. Oncol..

[15] Travis W.D., Brambilla E., Rami-Porta R., Vallières E., Tsuboi M., Rusch V., Goldstraw P. (2008). Visceral pleural invasion: Pathologic criteria and use of elastic stains: Proposal for the 7th edition of the TNM classification for lung cancer. J. Thorac. Oncol..

[16] Mauguen A., Pignon J.-P., Burdett S., Domerg C., Fisher D., Paulus R., Mandrekar S.J., Belani C.P., Shepherd F.A., Eisen T. (2013). Surrogate endpoints for overall survival in chemotherapy and radiotherapy trials in operable and locally advanced lung cancer: A re-analysis of meta-analyses of individual patients’ data. Lancet Oncol..

[17] Wolff R.F., Moons K.G.M., Riley R.D., Whiting P.F., Westwood M., Collins G.S., Reitsma J.B., Kleijnen J., Mallett S. (2019). PROBAST: A Tool to Assess the Risk of Bias and Applicability of Prediction Model Studies. Ann. Intern. Med..

[18] DeLong E.R., DeLong D.M., Clarke-Pearson D.L. (1988). Comparing the areas under two or more correlated receiver operating characteristic curves: A nonparametric approach. Biometrics.

[19] Travis W.D., Brambilla E., Nicholson A.G., Yatabe Y., Austin J.H.M., Beasley M.B., Chirieac L.R., Dacic S., Duhig E., Flieder D.B. (2015). The 2015 World Health Organization Classification of Lung Tumors: Impact of Genetic, Clinical and Radiologic Advances Since the 2004 Classification. J. Thorac. Oncol..

[20] Heinze G., Schemper M. (2001). A Solution to the Problem of Monotone Likelihood in Cox Regression. Biometrics.

[21] Luo W., Rao K., Chen H., Hu C., Liu S., Wang J., Zhang D., Chen L. (2026). Predicting Overall Survival in Patients With Multiple Primary Lung Cancer: Nomogram Development and Validation Study. JMIR Cancer.

[22] Ji Y., Zhao Q., Liu Y., Qiu B., Bai G., Ai S., Feng W., Yuan L., Wang X., Rong L. (2024). Establishment of a survival predictive model for patients with two synchronous multiple primary lung cancers: A multicenter cohort analysis. Transl. Lung Cancer Res..

[23] Song C.-K., Guo Z.-X., Shen X.-Y., Wang Y.-J., Wang Q.-W., Yu D.-H., Chen C., Liu X.-P., Huang J.-Y., Li S. (2020). Prognostic Factors Analysis and Nomogram Construction of Dual Primary Lung Cancer: A Population Study. BioMed Res. Int..

[24] Shiono S., Yanagawa N. (2016). Spread through air spaces is a predictive factor of recurrence and a prognostic factor in stage I lung adenocarcinoma. Interact. Cardiovasc. Thorac. Surg..

[25] Liu H., Yin Q., Yang G., Qie P. (2019). Prognostic Impact of Tumor Spread Through Air Spaces in Non-small Cell Lung Cancers: A Meta-Analysis Including 3564 Patients. Pathol. Oncol. Res..

[26] Yang Y., Lin H., Shi L., Yang X., Hu M., Tong L., Liu Z., Yu J., Lu B. (2025). The characteristics and prognoses of 72 postoperative synchronous multiple primary lung cancer patients. World J. Surg. Oncol..

[27] Zhang H., Chen L., Mao F., Li J., Hamaji M., Shimada Y., Koo C.W., Song Z., Song L., Lu S. (2024). Long-term prognosis analysis of surgical therapy for bilateral synchronous multiple primary lung cancer: A follow-up of 293 cases. J. Thorac. Dis..

[28] Xie H., Dou S., Huang X., Wen Y., Yang L. (2024). The effect of spread through air spaces on postoperative recurrence-free survival in patients with multiple primary lung cancers. World J. Surg. Oncol..

[29] Mansfield A.S., Murphy S.J., Peikert T., Yi E.S., Vasmatzis G., Wigle D.A., Aubry M.C. (2016). Heterogeneity of Programmed Cell Death Ligand 1 Expression in Multifocal Lung Cancer. Clin. Cancer Res..

[30] Heinze G., Dunkler D. (2008). Avoiding infinite estimates of time-dependent effects in small-sample survival studies. Stat. Med..

