# Supplementary material for: Paired Inter-Lesion Invasiveness and Recurrence Risk in Synchronous Multiple Primary Lung Adenocarcinoma: Focus on the IAC-Dominant Subgroup
Source: J Clin Med. 2026 Sep 20;15(18):7317. doi: 10.3390/jcm15187317 (PMC13607963; doi:10.3390/jcm15187317)
Supplement: Supplementary file 1 [file jcm-15-07317-s001.zip › jcm-4518351-supplementary.pdf]

Supplementary Table S1. Cochran–Armitage trend tests for Lesion 1 aggressiveness markers across the WHO-defined paired invasiveness score.

Marker	N	Positive, n	Cochran–Armitage Z	*P* (Trend)
PD-L1 TPS ≥1%	92	38	3.75	**<0.001**
Ki67 ≥10%	97	33	3.81	**<0.001**
Ki67 ≥20%	97	14	2.32	**0.020**
STAS positive	198	11	4.40	**<0.001**
Pleural invasion	198	5	3.05	**0.002**
Vascular invasion	198	2	1.59	0.113

The Cochran–Armitage test used a pre-specified WHO-based score, calculated as the sum of the two lesion-level invasiveness grades (AIS = 0, MIA = 1, IAC = 2). Thus, score 0 = AIS–AIS; score 1 = AIS–MIA; score 2 = MIA–MIA or AIS–IAC; score 3 = MIA–IAC; and score 4 = IAC–IAC. Positive Z values indicate increasing marker positivity with increasing score. The denominator for PD-L1 and Ki67 is the number with available Lesion 1 immunohistochemical data; the denominator for STAS, pleural invasion, and vascular invasion is the number with available pathological data. Abbreviations: PD-L1, programmed death-ligand 1; TPS, tumor proportion score; STAS, spread through air spaces.

Supplementary Table S2. Baseline characteristics of the IAC-dominant subset (n = 91).

Variable	Total (n = 91)	IAC + IAC (n = 34)	IAC + AIS/MIA (n = 57)	*P*
**Demographics**				
Age, years	62 [52–68]	63 [56–69]	58 [50–67]	0.187
**Gender**				0.118
Female	58/91 (63.7%)	18/34 (52.9%)	40/57 (70.2%)	
Male	33/91 (36.3%)	16/34 (47.1%)	17/57 (29.8%)	
**Smoking history**				0.381
Yes	39/91 (42.9%)	17/34 (50.0%)	22/57 (38.6%)	
No	52/91 (57.1%)	17/34 (50.0%)	35/57 (61.4%)	
**Tumor characteristics**				
Lesion 1 size, mm	12 [9–17]	12 [9–18]	12 [10–15]	0.665
Lesion 2 size, mm	6 [5–9]	8 [6–12]	5 [4–8]	**<0.001**
**Surgical procedure (dominant lesion)**				**0.630**
Lobectomy (dominant)	66/91 (72.5%)	26/34 (76.5%)	40/57 (70.2%)	
Sublobar (dominant)	25/91 (27.5%)	8/34 (23.5%)	17/57 (29.8%)	
**Detailed procedure**				**<0.001**
Lobectomy	34/91 (37.4%)	3/34 (8.8%)	31/57 (54.3%)	
Sublobar resection	8/91 (8.8%)	0/34 (0.0%)	8/57 (14.0%)	
Lobectomy + sublobar	32/91 (35.2%)	23/34 (67.6%)	9/57 (15.8%)	
Sublobar + sublobar	17/91 (18.7%)	8/34 (23.5%)	9/57 (15.8%)	
**Same lobe**				**<0.001**
Yes	42/91 (46.2%)	3/34 (8.8%)	39/57 (68.4%)	
No	49/91 (53.8%)	31/34 (91.2%)	18/57 (31.6%)	
Lesion count	2 [2–2]	2 [2–2]	2 [2–2]	0.164
LN N1 positive, n/N (%)	6/91 (6.6%)	3/34 (8.8%)	3/57 (5.3%)	0.668
**Stage**				0.186
IA1	32/91 (35.2%)	13/34 (38.2%)	19/57 (33.3%)	
IA2	41/91 (45.1%)	12/34 (35.3%)	29/57 (50.9%)	
IA3	9/91 (9.9%)	3/34 (8.8%)	6/57 (10.5%)	
IB	3/91 (3.3%)	3/34 (8.8%)	0/57 (0.0%)	
IIA	3/91 (3.3%)	1/34 (2.9%)	2/57 (3.5%)	
IIB	3/91 (3.3%)	2/34 (5.9%)	1/57 (1.8%)	
**Lesion 1 T stage**				0.085
Tis	0/91 (0.0%)	0/34 (0.0%)	0/57 (0.0%)	

T1mi	0/91 (0.0%)	0/34 (0.0%)	0/57 (0.0%)	
T1a	33/91 (36.3%)	13/34 (38.2%)	20/57 (35.1%)	
T1b	41/91 (45.1%)	12/34 (35.3%)	29/57 (50.9%)	
T1c	11/91 (12.1%)	4/34 (11.8%)	7/57 (12.3%)	
T2a	6/91 (6.6%)	5/34 (14.7%)	1/57 (1.8%)	
**Lesion 2 T stage**				**<0.001**
Tis	17/91 (18.7%)	0/34 (0.0%)	17/57 (29.8%)	
T1mi	40/91 (44.0%)	0/34 (0.0%)	40/57 (70.2%)	
T1a	24/91 (26.4%)	24/34 (70.6%)	0/57 (0.0%)	
T1b	9/91 (9.9%)	9/34 (26.5%)	0/57 (0.0%)	
T1c	1/91 (1.1%)	1/34 (2.9%)	0/57 (0.0%)	
T2a	0/91 (0.0%)	0/34 (0.0%)	0/57 (0.0%)	
**Lesion 1 location**				0.112
Right upper lobe	32/91 (35.2%)	13/34 (38.2%)	19/57 (33.3%)	
Right middle lobe	6/91 (6.6%)	4/34 (11.8%)	2/57 (3.5%)	
Right lower lobe	18/91 (19.8%)	3/34 (8.8%)	15/57 (26.3%)	
Left upper lobe	23/91 (25.3%)	11/34 (32.4%)	12/57 (21.1%)	
Left lower lobe	12/91 (13.2%)	3/34 (8.8%)	9/57 (15.8%)	
**Lesion 2 location**				0.789
Right upper lobe	36/91 (39.6%)	11/34 (32.4%)	25/57 (43.9%)	
Right middle lobe	10/91 (11.0%)	5/34 (14.7%)	5/57 (8.8%)	
Right lower lobe	10/91 (11.0%)	4/34 (11.8%)	6/57 (10.5%)	
Left upper lobe	21/91 (23.1%)	9/34 (26.5%)	12/57 (21.1%)	
Left lower lobe	14/91 (15.4%)	5/34 (14.7%)	9/57 (15.8%)	
**Pathology**				
**Lesion 1 pathology**				—
IAC	91/91 (100.0%)	34/34 (100.0%)	57/57 (100.0%)	
**Lesion 2 pathology**				**<0.001**
IAC	34/91 (37.4%)	34/34 (100.0%)	0/57 (0.0%)	
MIA	40/91 (44.0%)	0/34 (0.0%)	40/57 (70.2%)	
AIS	17/91 (18.7%)	0/34 (0.0%)	17/57 (29.8%)	
**Lesion 1 aggressive features**				
STAS positive	11/91 (12.1%)	8/34 (23.5%)	3/57 (5.3%)	**0.017**
Pleural invasion	5/91 (5.5%)	4/34 (11.8%)	1/57 (1.8%)	0.063
Vascular invasion	2/91 (2.2%)	1/34 (2.9%)	1/57 (1.8%)	1.000
**Immunohistochemistry (Lesion 1)**				
Ki67, %	5.0 [4.5–10.0]	10.0 [5.0–17.5]	5.0 [2.0–10.0]	**0.032**
PD-L1 TPS, %	0.9 [0.0–2.8]	1.0 [1.0–6.5]	0.8 [0.0–1.0]	**<0.001**
Ki67 ≥10%	30/68 (44.1%)	16/27 (59.3%)	14/41 (34.1%)	0.050
Ki67 ≥20%	12/68 (17.6%)	7/27 (25.9%)	5/41 (12.2%)	0.197
PD-L1 TPS ≥1%	35/70 (50.0%)	21/27 (77.8%)	14/43 (32.6%)	**<0.001**

Note: Bold *p* values indicate $P < 0.05$. Continuous variables are presented as median [IQR] and compared using the Mann–Whitney U test. Categorical variables are compared using Fisher's exact test or the χ^2 test, as appropriate. Surgical procedure was categorized according to the extent of resection for the dominant lesion (Lesion 1). "Lobectomy (dominant)" includes lobectomy alone and lobectomy plus sublobar resection for the secondary lesion; "Sublobar (dominant)" includes sublobar resection alone and sublobar resection for both lesions. Detailed four-category classification is also shown. Among the IAC + IAC pairs, 3 cases from the same lobe were confirmed as clonally independent by NGS; 31 cases from different lobes were diagnosed as independent primary tumors according to ACCP criteria. Abbreviations: IAC, invasive adenocarcinoma; MIA, minimally invasive adenocarcinoma; AIS, adenocarcinoma in situ; PD-L1, programmed death-ligand 1; TPS, tumor proportion score; STAS, spread through air spaces; NGS, next-generation sequencing; ACCP, American College of Chest Physicians.
